# Benzo[b]fluoranthene Impairs Mouse Oocyte Maturation *via* Inducing the Apoptosis

**DOI:** 10.3389/fphar.2020.01226

**Published:** 2020-08-28

**Authors:** Jing Guo, Jiayu Huang, Liqun Zhang, Chong Li, Yinhua Qin, Weiwei Liu, Jingyu Li, Guoning Huang

**Affiliations:** ^1^Chongqing Key Laboratory of Human Embryo Engineering, Chongqing Reproductive and Genetics Institute, Chongqing Health Center for Women and Children, Chongqing, China; ^2^Department of Obstetrics and Gynecology, Union Hospital, Tongji Medical College, Huazhong University of Science and Technology, Wuhan, China; ^3^Department of Obstetrics and Gynecology, The First Hospital of Jilin University, Changchun, China

**Keywords:** oocyte maturation, mitochondria, AMPK, DNA damage, apoptosis

## Abstract

Benzo[b]fluoranthene (BbF) is one of the main pollutants of polycyclic aromatic hydrocarbons (PAHs), which are generated from organic materials combustion and diesel exhaust. It has been reported that after maternal exposure, BbF crosses the placental barrier, leading to offspring defects. However, the effect of BbF on the female reproductive system, especially on oocyte maturation has not been studied. To elucidate the effect and precise mechanism of BbF on oocyte maturation, nuclear, and cytoplasm maturation were evaluated after exposing mouse oocytes to different concentrations of BbF. Results showed that BbF exposure shows no effect on the meiotic progression, but it caused defects on nuclear maturation *via* impairment on chromosome alignment. In addition, the treatment of BbF displayed the defects on the cytoplasmic maturation by leading to the mitochondrial dysfunction, DNA damage accumulation, early apoptosis and the loss of H3K4me3. To investigate the mechanism, we found that BbF impaired the oocyte maturation *via* the AMPK pathway. BbF exposure caused the phosphorylation of AMPK, which cause the DNA damage accumulation and apoptotic incidence. Taken together, our results demonstrated that BbF exposure impaired the mouse oocyte maturation due to mitochondrial dysfunction and early apoptosis.

## Introduction

In recent years, environmental pollutions attracted people’s attention because of its potential risk to human health. Polycyclic aromatic hydrocarbons (PAHs) are a class of ubiquitous air pollutant compounds all over the world which are produced by incomplete organic materials combustion and diesel exhaust (Mesquita et al., 2014). PAHs are also the main pollutants of the haze. PAHs adsorb on suspended particulate matter (PM), which is distributed through blood circulation into the whole body. PM2.5 (aerodynamic diameter ≤ 2.5 µm) is recognized to produce adverse health effects (Abbas et al., 2019). Previous studies have reported PAHs reproductive toxicity by crossing the placental barrier, which causes offspring defects, such as pre-term birth, low birth weight, and intrauterine growth retardation (Drwal et al., 2019).

Benzo[b]fluoranthene (BbF) is a typical and major PAH compound with five fused benzene rings aromatic hydrocarbon generated from incomplete combustion or pyrolysis of organic materials, which plays an important role in air pollution (Castano-Vinyals et al., 2004). BbF is present in the atmosphere, land, water, and foods. Furthermore, it is recognized as potentially carcinogenic to humans. In China, BbF concentration ranges from 2 to 21 ng/m^3^, which represents the highest concentration of fluoranthene in China (Ma et al., 2018). Female work under air-polluted environment, including petrochemical industry and major traffic roads, causing abnormal menstrual cycle, hormone levels and a high risk of infertility (Thurston et al., 2000; Tomei et al., 2006; Mahalingaiah et al., 2016). Exposure to BbF increases the risk of cancer by ingestion, inhalation, and dermal contact (Soltani et al., 2015), induces lung tumors and kidney disease (Mass et al., 1996; Zhang et al., 2020). BbF exposure also displayed reproduction toxicity. The ovary is the major target organ for BbF. In addition, BbF can be detected in human milk, maternal, and cord serum (Del Bubba et al., 2005; Yin et al., 2017). In this regard, a previous study found that pregnant mice were orally exposed to BbF, sperm concentration, and quality of their male offspring were decreased significantly (Kim et al., 2011). So the toxicity of BbF can be passed to the offspring. Most studies focused on correlation of PAH chemicals and pregnancy outcomes or reproductive systems. However, only a few of them investigated the effect of PAHs on female reproductive function, especially on oocyte maturation. As known that oocyte maturation is a complex process that arrests at the MII stage until fertilization. During meiosis, oocytes are sensitive to exogenous toxic agents, causing dysfunction of nuclear and cytoplasm maturation, which decreases fertilization and embryonic development potential. Among PAH compounds, benzo[a]pyrene (BaP) and Benzo[ghi]perylene (B[ghi]P) displayed the toxicology on the oocyte maturation (Miao et al., 2018; Li et al., 2019). It is known that BbF is harmful to male offspring, however, its effect and exact mechanism on oocyte maturation have not been investigated.

In the present study, we detect the toxic effect of BbF on the quality of mouse oocytes. Results showed that BbF exposure did not affect the oocyte maturation process, but perturbed the chromosome alignment. In addition, it caused mitochondrial dysfunction by decreasing the amount of mitochondria and membrane potential. BbF exposure led to AMPK phosphorylation, which induced DNA damage and early apoptosis, and loss of H3K3me3.

## Materials and Methods

### Animals

In this study, we used 6- to 8-week-old ICR female mice. One hundred mice were subjected to superovulation. Animal care and handling were conducted in accordance with the policies regarding the care and use of animals, issued by the ethics committee of Jilin University (SY201903007).

### Chemicals

Chemicals were purchased from the Sigma (St Louis, MO, USA) unless otherwise indicated.

### Oocyte Collection and Culture

Female mice were superovulated by an intraperitoneal injection of 10 IU pregnant mare serum gonadotropin, and killed by cervical dislocation. Germinal vesicle (GV) oocytes were collected in M2 medium, then cultured in M16 medium supplemented with or without BbF under mineral oil in a cell‐culture dish at 37°C for 12 h in a humidified atmosphere of 5% CO2 and 95% air. After 12 h of maturation, MII oocytes were collected for subsequent analysis. The medium and other oocytes were treated as medical waste to handle.

### BbF Treatment

BbF was dissolved in dimethylsulphoxide and diluted to a final concentration of 8, 20 or 40 μM with M16 medium, respectively. The concentration of solvent in the medium was less than 0.1%. All operations were carried out in biosafety cabinet. This study was adhered to standard biosecurity and institutional safety procedures.

### Immunofluorescence and Confocal Microscopy

Oocytes were fixed in the 4% paraformaldehyde in PBS containing 0.1% polyvinyl alcohol (PVA) for 20 min at room temperature. Oocytes were washed in PBS-PVA, then permeabilized in the PBS-PVA (0.5% Triton X-100) for 1 h. After blocking with 3% bovine serum albumin (BSA) solution, oocytes were incubated with anti-α-tubulin FITC antibody, H3K4me3 (Cell Signaling Technology, Danvers, MA,USA) or p-AMPK(Sangon Biotech, Shanghai, China) overnight at 4°C. Oocytes were counterstained with Hoechst 33342 for 15 min. Finally, oocytes were mounted on a glass slide and examined using an a laser‐scanning confocal microscope (Leica TCS SP8)

Mito-Tracker Red CMXRos (Beyotime, Shanghai, China) was used to detect the mitochondria. Oocytes were incubated with Mito-tracker for 20 min at 37°C. Mitochondrial membrane potential was evaluated with JC-1 assay kit (Beyotime, Shanghai, China). Annexin-V (ThermoFisher Scientific, Waltham, MA, USA) was used to detect the early apoptosis. All the pictures were captured with the same settings. The fluorescence intensity was analyzed using Image J software.

### Chromosome Spread

MII oocytes were exposed to the Tyrode’s buffer for several seconds to remove the zona pellucida. After recovery in M2 medium, oocytes were fixed in a drop of 1% paraformaldehyde containing 0.15% Triton X-100 and 3 mM dithiothreitol at 4°C overnight. Chromosomes were washed 3 times with PBS-PVA, then blocking for 1 h. After blocking, chromosomes were stained with Hoechst 33342 and anti-crest antibody (Antibodies Incorporated, Davis, CA, USA), and examined under a laser scanning confocal microscope.

### Quantitative Real-Time PCR

Total RNA was extracted from oocytes, using the Arcturus PicoPure RNA isolation Kit, according to manufacturer’s instructions (Thermo Fisher Scientific, Waltham, MA, USA), followed by reverse transcription and qRT-PCR, using the PrimeScript RT Master Mix (Takara, Dalian, China) and the SYBR Green qRT-PCR master mix (Takara, Dalian, China) respectively. The amplification cycles were as follows: 95°C for 3 min followed by 40 cycles of 95°C for 15 s, 60°C for 30 s, and 72°C for 20 s, and a final extension at 72°C for 5 min. Relative gene expression was normalized to internal Gapdh mRNA levels.

### Western Blot Analysis

Mouse oocytes were lysed in the RIPA lysis buffer with PMSF (Beyotime, Shanghai, China), and heated in 95°C for 5 min. Protein samples were separated to SDS-PAGE on 10% polyacrylamide gels, and subsequently transferred to PVDF membranes, blocked in 5% skim milk in TBS with 0.1% Tween 20 for 1 h at room temperature. The membranes were incubated overnight with the primary antibody for anti-pAMPK or anti-γ-H2AX (Cell Signaling Technology, Danvers, MA,USA). Membranes were washed 3 times in TBST, and incubated for 1 h with goat anti-rabbit IgG (Servicebio, Wuhan, China) at room temperature. Chemiluminescence was performed with ECL Plus (Servicebio, Wuhan, China) and signals were captured by Protein Simple imaging system.

### Statistical Analysis

Three independent experiments were conducted. At least thirty oocytes were used for analysis. The data were presented as mean ± SEM and analyzed with one-way analysis of variance using the software GraphPad Prism (version 6.01). P < 0.05 was considered statistically significant.

## Results

### BbF Treatment Displayed No Effect on the Oocyte Maturation Progression

**Figure 1:**
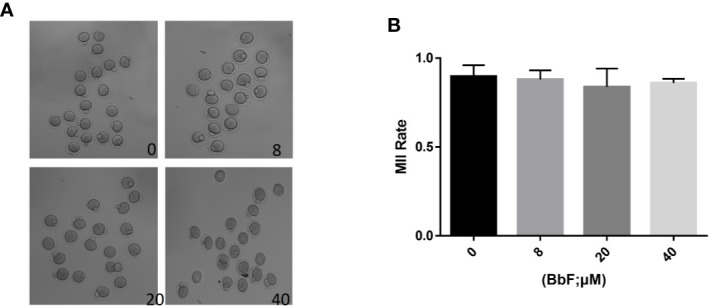
The effect of different concentrations of BbF on mouse oocyte maturation. **(A)** Representative images of oocyte maturation were shown in control and BbF exposed groups. **(B)** The rate of MII oocyte was quantified after culturing for 12 h with different concentrations of BbF.

To detect the BbF toxic effect on the oocyte maturation progression, different concentration of BbF (0, 8, 20, 40 µM) was added to the medium and the polar body extrusion was observed. Results showed that there was no difference on the oocyte maturation rate (**Figure 1A**). As shown in **Figure 1B**, BbF at all concentrations tested did not alter mouse oocyte maturation progression, as compared with the control group (81.9 ± 2.8%, 82.8 ± 3.9%, 86.0 ± 0.1% vs 89.6 ± 2.9%).

### BbF Treatment Caused the Chromosome Misalignment and Increased the Aneuploidy

**Figure 2:**
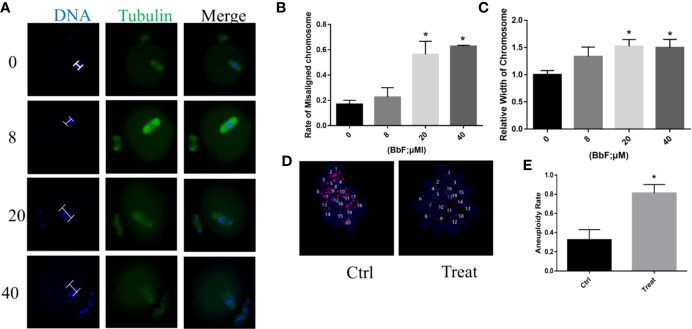
The effect of BbF on chromosome alignment in mouse oocytes. **(A)** Representative images of chromosome alignment in different concentrations of mouse oocytes. **(B)** The proportion of chromosome misalignment in control and BbF exposed groups. **(C)** The relative width of chromosome was shown after BbF exposure. **(D)** Representative images of euploid and aneuploid oocytes in control and BbF-treated groups. **(E)** The rate of aneuploidy was recorded in control and BbF-treated groups.*p < 0.05.

Although BbF treatment had no effect on oocyte maturation rate, the normal spindle and chromosome alignment were evaluated. When the oocytes exposed to 8 µM BbF, the rate of chromosome misalignment was not significantly higher than the control. However, concentration of 20, 40 µM BbF increased the rate of chromosome misalignment significantly (**Figures 2A, B**). The length of chromosome was also increased (**Figure 2C**). The chromosome spread was performed to check the aneuploidy, we found that the percentage of aneuploidy was increased after BbF exposure (**Figures 2D, E**). These findings demonstrated that BbF exposure leads to the chromosome misalignment and aneuploidy.

### BbF Exposure Reduced the Amount of Mitochondria

**Figure 3:**
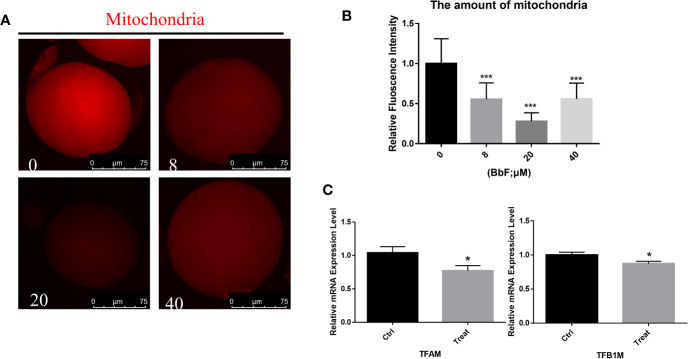
The effect of BbF on amount of mitochondria. **(A)** Representative images of amount of mitochondria after BbF exposure. **(B)** The relative fluorescence intensity of mitochondrion signals was shown in different concentrations of BbF. **(C)** mRNA expression level of mitochondrial biogenesis-related genes in control and BbF-treated groups. *P<0.05, ***P < 0.001.

Because mitochondria are essential for cytoplasm maturation in oocyte maturation, total mitochondria were detected with immunostaining. As shown in **Figure 3A**, the amount of mitochondria was reduced. The relative fluorescence intensity was also analyzed, the result also displayed that the amount of mitochondria was decreased significantly between the BbF exposure and control group (**Figure 3B**). Next, the mitochondrial biogenesis biomarkers was detected. The BbF exposure significantly decreased the mRNA expression of *Tfam* and *Tfb1*. These results suggest that BbF induced a decreased amount of mitochondria which is contributed to the reduction in mitochondrial biogenesis.

### BbF Caused the Mitochondrial Dysfunction

**Figure 4:**
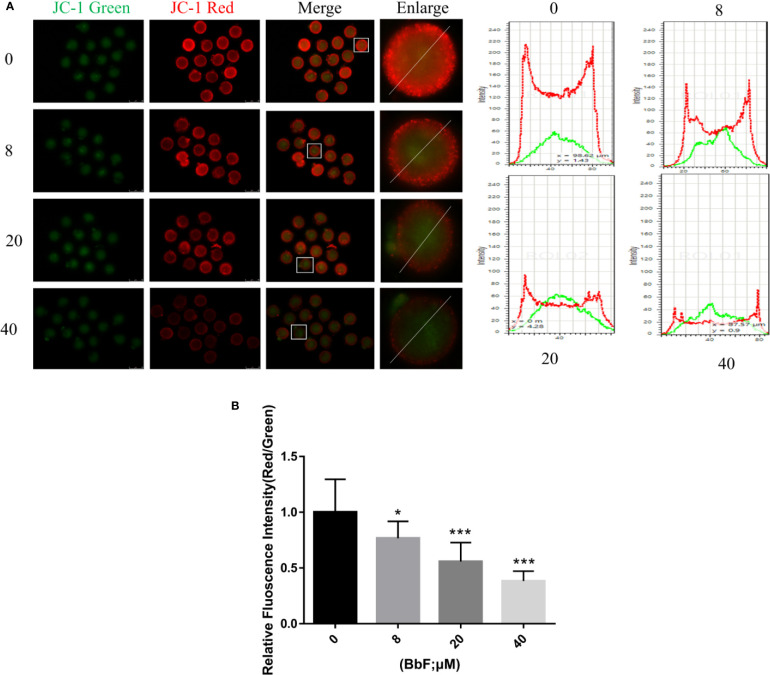
The effect of BbF on mitochondria membrane potential. **(A)** Representative images of JC-1 staining and plot profile analysis in control and BbF-treated groups. **(B)** The relative fluorescence intensity of JC-1 staining in oocytes. *p < 0.05, ***P < 0.001.

To further explore the mechanism underlying the effect of BbF on oocyte maturation, the mitochondrial membrane potential was checked. After oocytes were exposed to BbF, representative images of mitochondrial membrane potential are shown in **Figure 4A**. The results showed that the value of mitochondrial membrane potential which was evaluated by the rate of red/green fluorescence intensity was significantly decreased upon BbF exposure during the oocyte maturation period (**Figure 4B**). Therefore, the BbF exposure causes the mitochondria dysfunction *via* the detection of mitochondrial membrane potential.

### BbF Exposure Leads to the Phosphorylation of AMPK

**Figure 5:**
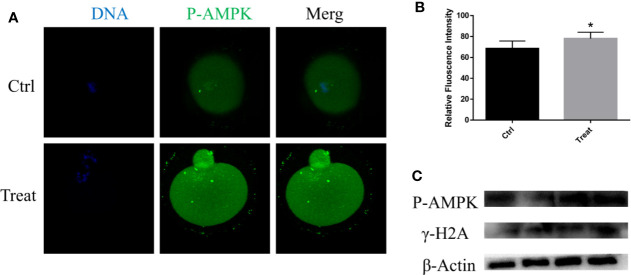
BbF induces DNA damage *via* the phosphorylation of AMPK. **(A)** Representative images of phosphorylated-AMPK in control and BbF-treated groups. **(B)** The fluorescence intensity of p-AMPK in control and BbF-treated groups. **(C)** Western blotting result of p-AMPK, γ-H2A and β-Actin in control and BbF-treated groups. *P<0.05.

Mitochondria are important organelles for ATP generation, and AMPK is considered as a metabolism regulator to balance ATP generation (Herzig and Shaw, 2018). The activation of AMPK was detected, the results showed that the p-AMPK was increased after BbF treatment (**Figures 5A, B**). The activation of AMPK cause the DNA damage accumulation (**Figure 5C**). These results showed that phosphorylation of AMPK results in the DNA damage accumulation and mitochondrial dysfunction due to the BbF exposure.

### BbF Exposure Results in the Early Apoptosis

**Figure 6:**
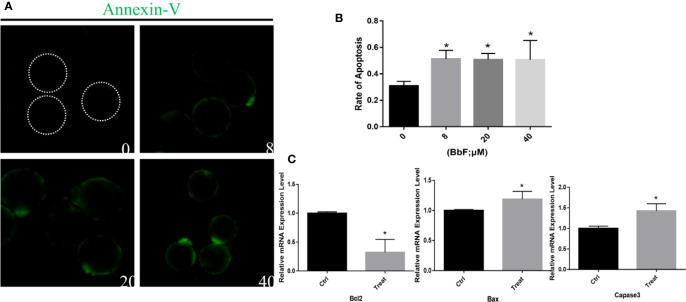
The effect of BbF on early apoptosis in BbF-exposed mouse oocytes. **(A)** Representative images of apoptotic oocytes in different concentrations of BbF exposure groups. **(B)** The rate of early apoptosis was shown. Oocytes were cultured for 12 h with different concentrations of BbF. **(C)** Relative mRNA expression level of apoptosis related genes, Bax, Bcl2, Caspase3. *p < 0.05.

BbF exposure leads to mitochondrial dysfunction and AMPK activation, which initiate early apoptosis, therefore we hypothesized that BbF induced the initiation of early apoptosis. To confirm this hypothesis, apoptosis was assessed by the Annexin-V staining. Results showed that more Annexin-V positive oocytes were observed after BbF exposure (**Figure 6A**). In addition, the rate of apoptosis was dramatically higher than the control group (**Figure 6B**). To further verify the function of BbF on the apoptosis, the anti-apoptotic and pro-apoptotic genes *Bcl2*, *Bax*, and *Caspase3* expression was determined, the BbF treatment decreased the mRNA expression level of *Bcl2* and increased the expression of *Bax* and *Caspase3* (**Figure 6C**). Therefore, the BbF exposure induced the apoptosis *via* the regulation of apoptosis related genes expression in the oocyte maturation period.

### BbF Exposure Causes the Loss of H3K4me3

**Figure 7:**
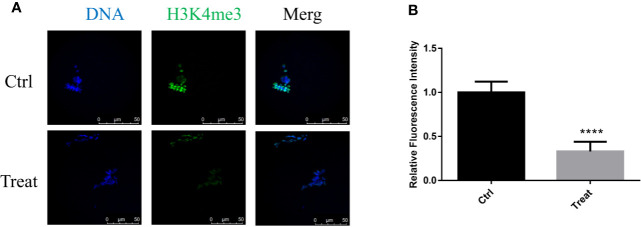
The effect of BbF on histone H3K4me3 in BbF-exposed mouse oocytes. **(A)** Representative images of histone H3K4me3 in different concentrations of BbF exposure groups. **(B)** Quantitative analysis of H3K4me3 fluorescence intensity in different concentrations of BbF exposure groups. ****P < 0.001.

H3K4me3 plays an important role in oocyte epigenetic maturation, and regulates the global transcription activity (Yu et al., 2017). It was essential to demonstrate whether BbF reduces histones methylation patterns. Analysis of H3K4me3 in control and BbF exposure groups showed that H3K4me3 decreased after BbF exposure (**Figure 7A**). The decrease of H3K4me3 relative fluorescence intensity confirmed the effect of BbF on the H3K4 methylation pattern (**Figure 7B**). Therefore, our results suggest that the BbF exposure induces the loss of histone modification.

## Discussion

Recently, air pollution attracts people’s attention. As the main source of air pollution, PAH is implicated in the reproductive disorders. As shown in previous studies, the effects of PAH on reproductive system include fertility, pregnancy, and ovarian physiology (Bolden et al., 2017). Maternal exposure to PAHs prior to pregnancy and/or during lactation compromises reproductive potential of female mice, diminishing ovarian reserve and causing DNA damage (Jurisicova et al., 2007; Ogliari et al., 2013). Air pollution has a potentially toxic effect in the ovary. Since PAHs exposure always involves complex mixtures, we were unable to determine which of their components was associated with this effect. The most frequently studied individual compound was BaP. BbF is one of the main composition of PAH (Kim et al., 2011). There is no reports about the toxic impact of BbF on the female reproduction system, especially on oocyte maturation.

In the present study, we investigated whether BbF exposure contributes to the failure of oocyte developmental competence. First, the meiotic progression was detected after exposure to BbF. However, there was no difference in the oocyte maturation rate, suggesting that BbF showed no effect on polar body extrusion. Further examination found that BbF exposure resulted in dispersed and misaligned chromosomes. In addition, BbF addition led to generation of aneuploidy oocytes, which is known to impair fertilization and embryo developmental potential (MacLennan et al., 2015). Therefore, BbF exposure may affect chromosome arrangement to generate aneuploidy. The results also showed that BbF treatment caused defects on cytoplasmic maturation, leading to mitochondrial dysfunction, DNA damage accumulation, early apoptosis, and loss of H3K4me3. Although oocyte maturation progression was not affected by BbF, its toxicity may decrease the embryonic development potential. Previous studies also found that other individual component of PAH was toxic to oocytes; Bap and B[ghi]P exposure caused nuclear and cytoplasmic maturation failure, *via* impairing the meiotic apparatus (Miao et al., 2018; Zhang et al., 2018; Li et al., 2019). These studies demonstrated that PAHs showed a similar effect on oocyte maturation. We found that BbF exposure also result in the failure of nuclear and cytoplasmic maturation in accordance with others. However, BbF exposure did not affect polar body extrusion, which might be due to the lower BbF concentration used in this study. In addition, the BbF toxicity may be less than others. Therefore, the BbF has no dramatically toxic effect as well as others.

Recent studies report that BbF concentration is from 2 to 21 ng/m^3^ in China (Ma et al., 2018). The lowest concentration of BbF used in this study was 8 μM (2 μg/mL), which is practically absent or rarely encountered in the actual living environment. We predicted that exposure to high concentrations of BbF would have significant effects on oocyte maturation, and experimentally explored the underlying mechanism. Previous studies showed that the lowest effective concentrations of BbF ranged from 1 μM to 25 μM, depending on cell types, which were used in the present study (Tarantini et al., 2011; Meldrum et al., 2016; Zhang et al., 2020).

Previous studies showed that the concentration of PAH component is from 5 to 200 ng/cigarette. The concentration of PAH component is only 1.32 ± 0.68 ng/ml in the follicular fluid of smoking woman. However, this concentration can influence women fertility. This concentration of PAH also decreased mouse follicle growth (Neal et al., 2007; Sadeu and Foster, 2011). The PAH can influence directly on the ovary, the ovary is the target organ affected by PAH.

Cytoplasm maturation is a symbol for the oocyte maturation, and an important determinant of embryo competence (Liang et al., 2018). Mitochondria are essential organelles in oocyte cytoplasm maturation because of their important roles in ATP production (Wang et al., 2009). In addition, mitochondria dysfunction is associated with defective oocyte maturation, involving apoptosis (Tiwari et al., 2015; Zhang et al., 2019). Therefore, mitochondria function is an important indicator of oocyte cytoplasm maturation. The amount and function of mitochondria were explored in BbF-exposed oocytes. Results revealed that the amount of mitochondria shown by fluorescence intensity significantly decreased. A massive increase in the number of mitochondria is critical during oocyte cytoplasmic maturation (Wang et al., 2009). BbF may then compromise mitochondria functionality and impair oocyte quality. To confirm this effect, the mitochondria-related genes were defined. *Tfam* and *Tfb1*, which are transcription factors to stimulating mitochondria biogenesis, decreased upon BbF exposure. BbF may cause damage to mitochondria biogenesis, leading to impairing oocyte cytoplasm maturation. Furthermore, alternations of mitochondria function were evaluated by measuring mitochondrial membrane potential. Mitochondrial membrane potential is a critical index for mitochondrial function. It is associated with ATP generation and induction of apoptosis (Liang et al., 2016). BbF exposed oocytes showed a decrease in mitochondrial membrane potential. These findings demonstrated that BbF destroyed mitochondria function in oocytes. The BbF compromise the oocyte maturation *via* the mitochondria damage.

One of the most important functions of AMPK is regulating metabolism, and previous studies revealed that AMPK is involved in mitochondrial health, including mitochondrial biogenesis and homeostasis (Herzig and Shaw, 2018). BbF exposure contributed to mitochondrial dysfunction *via* AMPK phosphorylation, which activates p53 and induces p21 upregulation to cause DNA damage (Zhang et al., 2010). We also found that BbF exposure caused DNA damage during oocyte maturation. The BbF exposure may induce the DNA damage, probably through AMPK phosphorylation, which contributes to decreasing oocyte quality.

Decrease of mitochondrial membrane potential and DNA damage induce apoptosis. Therefore, apoptosis was evaluated by the Annesin-V staining. We found that positive apoptotic oocytes increased after BbF exposure. *Bcl2*, *Bax*, and *Caspase3* are involved in the mitochondrial-mediated apoptosis pathway. Adverse factors would activate *Bax*, inhibit *Bcl2*, and release cytochrome c from mitochondria, which initiate the Caspase cascade activation to induce the apoptosis process (Elmore, 2007). Our results suggest that *Bcl2* expression decreased, but *Caspase3* increased after BbF exposure. These findings demonstrated that BbF induced mitochondrial dysfunction and accumulation of DNA damage, which further initiate apoptosis, impairing oocyte cytoplasm maturation.

Epigenetic status changes saliently during oocyte maturation in which epigenetic modifications are sensitive to environment (Ding et al., 2017; Wang et al., 2018). A major concern is that air pollution can disrupt epigenetic modifications in oocytes, which is demonstrated by previous studies showing that DNA methylation can lead to abnormal gene expression (Carre et al., 2017). Histone modification is one of the most important epigenetic modifications, which is important for oocyte maturation and embryonic development. The highly conserved epigenetic marker H3K4me3 is crucial for oocyte epigenetic maturation. H3K4 methylation status maintains transcription and triggers maternal-zygotic transition (Andreu-Vieyra et al., 2010; Yu et al., 2017). In addition, H3K4me3 is reprogrammed in early embryos (Zhang et al., 2016). Our results indicated that BbF exposure decreased H3K4me3 levels during oocyte maturation. However, the mechanism underlying the interplay between BbF and histone modification needs further investigation.

## Conclusion

**Figure 8:**
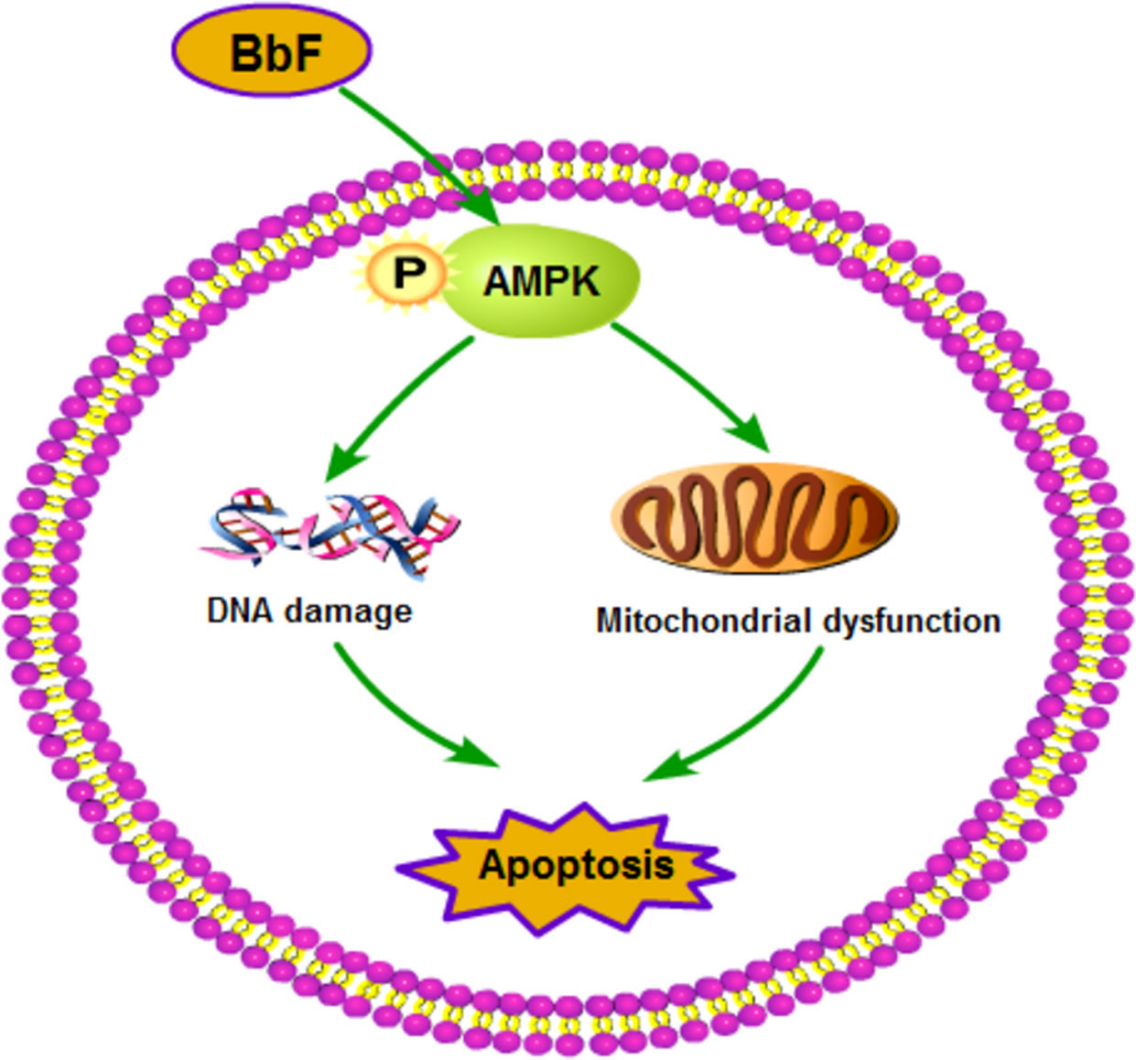
Summary of the toxicity effect of BbF on mouse oocyte maturation. The BbF exposure leads to the phosphorylation of AMPK to cause the mitochondrial dysfunction and DNA damage accumulation. Further, BbF exposure induces the early apoptosis due to the mitochondrial dysfunction and DNA damage.

In conclusion, our study demonstrates that BbF exposure compromise the mouse oocyte maturation. Oocytes exposing to BbF displays chromosome misalignment and aneuploidy. In addition, BbF phosphorylate AMPK to damage the DNA and mitochondria inducing the early apoptosis (**Figure 8**). Our research not only provided evidence for the toxic impact of BbF, but also deeply clarify the potential mechanism of BbF induced oocyte quality reduction.

## Data Availability Statement

All datasets generated for this study are included in the article/supplementary material.

## Ethics Statement

The animal study was reviewed and approved by the ethics committee of Jilin University.

## Author Contributions

JG, GH, and JL designed the research. LZ, CL, and YQ performed the experiments. WL and JG analyzed the data. JG, JH, GH, and JL wrote the manuscript. All authors contributed to the article and approved the submitted version.

## Funding

This study was supported by the Special fund for clinical research of Chinese Medical Association (17020430712, 17020440713) and Chongqing YuZhong Science Project (20170127).

## Conflict of Interest

The authors declare that the research was conducted in the absence of any commercial or financial relationships that could be construed as a potential conflict of interest.
